# A Rare Presentation of Encapsulated Left Ventricular Thrombus

**DOI:** 10.7759/cureus.70592

**Published:** 2024-10-01

**Authors:** Mohamed Elhassan, George Doos

**Affiliations:** 1 Cardiology, University Hospitals Birmingham, Birmingham, GBR

**Keywords:** cardiomyopathy, encapsulated thrombus, hyper coagulable state, left ventricular thrombus, mural thrombus

## Abstract

Left ventricular thrombus formation is not an uncommon complication. There are a variety of reasons for this, for example, myocardial infarction, aneurysm formation and hypercoagulability. This usually has different fates; the most serious of which is propagation and embolization causing distal organ dysfunction. Thrombus formation appearance on echocardiography can sometimes give an idea of whether this is acute, subacute or chronic. In this article, we present a rare case in which large thrombi were noted on transthoracic echocardiography to be fully encapsulated within the left ventricle. It is unclear whether this phenomenon represents chronicity or whether this has consequential clinical impacts of significance. To our knowledge, this has only rarely been reported in the literature. We present an in-depth discussion of the presentation along with reported postulated mechanisms that might have a role in encapsulation per se.

## Introduction

Left ventricular thrombus formation usually occurs as a complication of acute myocardial infarction or dilated cardiomyopathy [1]. It also occurs in patients with a hypercoagulable state. The development of intraventricular thrombosis relates to the components of Virchow’s triad, which include vascular endothelial damage. This can result from myocardial injury, hypercoagulability status that is usually attributable to various inflammatory processes, and lastly, stasis of blood resulting from left ventricular dysfunction [2]. Thus, left ventricular thrombus formation is very common in this context.

Left ventricular thrombi can be acute or chronic, with varying degrees of appearance on trans-thoracic echocardiography [3]. Non-laminated encapsulated mural thrombi have only rarely been reported and described in the literature. In the following text, we present an observed rare case where this is noted.

## Case presentation

We present a case of a 76-year-old male with a known past medical history of glaucoma and hypertension. He was brought into the emergency department as an alert call after he was found by neighbours lying on the floor for an unknown period. He was last seen 10 days ago. It appeared that he had had a long lie with a suspected fall, injuries to the head and various tissue loss on his torso. Upon arrival on the first day, he was noted to be critically unwell, confused, hypotensive and peripherally shut down. His vital signs showed the following: blood pressure (BP) 88/50, pulse rate (PR): 110/min, respiratory (RR) 22 cycles/min, temperature 34.9 °C, Glasgow Coma Scale of 14/15 and capillary blood glucose was 15 mmol/l. Although his airway was maintained, he was noted to have reduced air entry to both lung bases with no appreciable added sounds. He was catheterised but there was minimal urine output despite being on fluid resuscitation. On exposure, he was noted to have extensive areas of moisture damage/necrosis involving the trunk and limbs with a deep tissue injury to the back of his head. In addition, there was evidence of acute limb ischaemic changes on his toes with asymmetrical absent distal pulses (confirmed by bedside Doppler).

His blood tests (Table 1) revealed raised creatine kinase (CK), disproportionately high urea-to-creatinine (pre-renal) ratio, and raised sodium levels; otherwise, the full blood count and the rest of the electrolytes were largely unremarkable.

**Table 1:** Lab results

Investigation	Result	Normal reference range
Sodium level, blood (mmol/L)	150	135-140
Potassium level, blood (mmol/L)	4.4	3.5-4.9
Urea level, blood (mmol/L)	17	2.4-6.9
Creatinine level, blood (μmol/L)	92	63-133
High-sensitivity Troponin T level, serum (ng/L)	250 to 283 (serial)	0-16
Creatine kinase (CK) level, blood (Units/L)	22,450	20-190

His CXR showed cardiomegaly. The CT scan of his brain showed no evidence of acute intracranial pathology. A 12-lead ECG confirmed atrial fibrillation with some premature ventricular ectopic beats.

The patient was booked for a trauma series PAN-CT scan with contrast on the same day; this did not show any evidence of trauma. However, this revealed important cardiac findings that will be discussed later in this text.

As part of the emergency assessment, a focused assessment with sonography for trauma (FAST) extended ultrasound scan identified an abnormal left ventricular function with unclear echogenic structures. This triggered further assessment via a dedicated transthoracic echocardiography study. This showed large encapsulated multiple thrombi of high burden located in the LV apical region (Video 1). It also showed severe bi-ventricular systolic dysfunction with an ejection fraction of 25% measured via Simpson’s biplane method. The left ventricle was dilated with evidence of global hypokinesia. It also showed moderate mitral regurgitation with bi-atrial dilatation.

**Video 1:** Echocardiography - selected combined views from focused, left ventricular, apical four-chamber (A4C) with a sweep Three-chamber view (3CV) and two-chamber view (2CV) demonstrating encapsulated mural thrombi

As noted in Video 1, there are large, encapsulated, apical multiple thrombi. The foremost apical portion of these thrombi appears laminated while a few discrete thrombi appear non-laminated but confined within a membrane-like structure. The largest of the thrombi measured ~ 4.8 cm x 4 cm x 2.3 cm.

**Figure 1:**
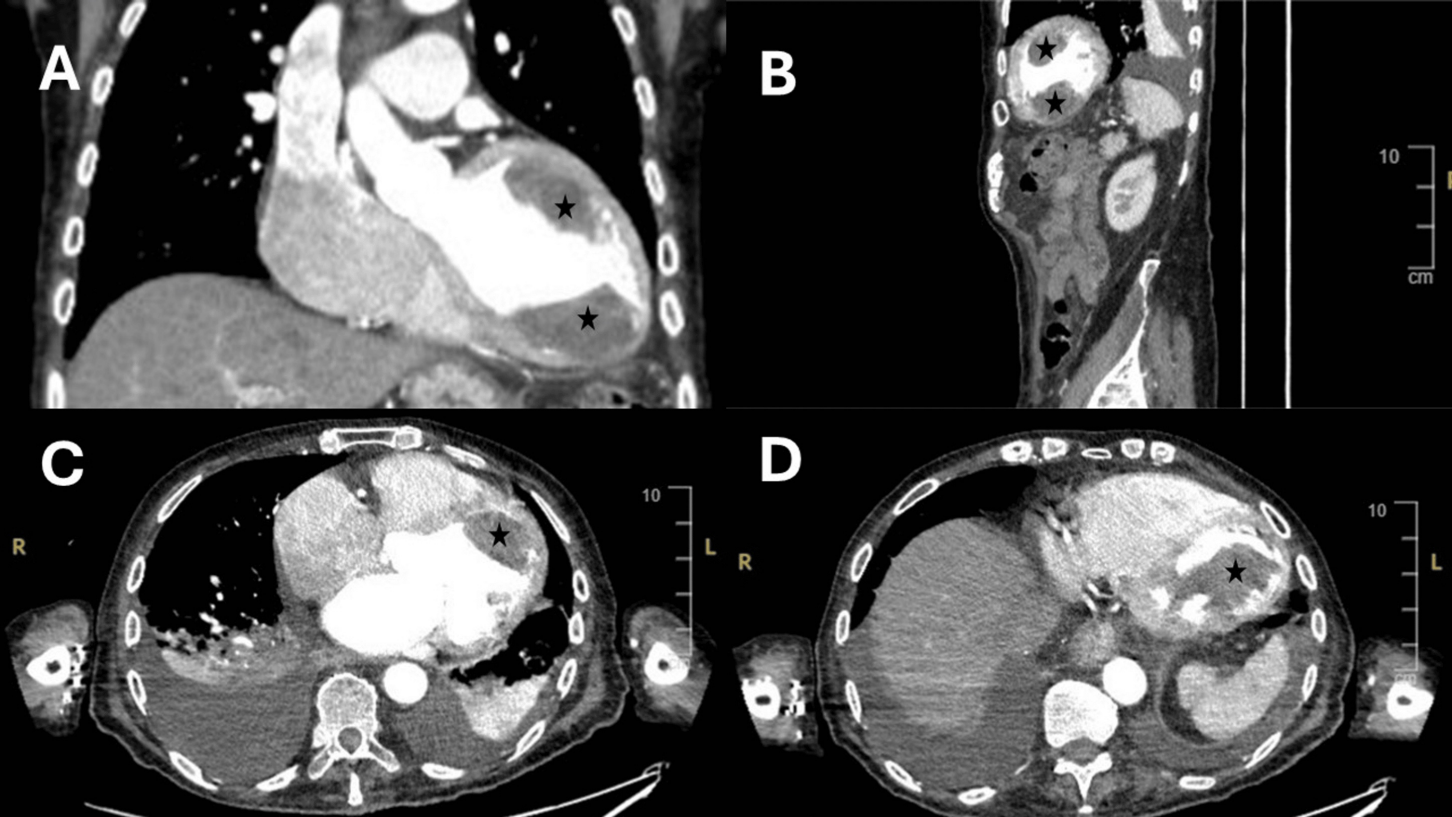
Selected CT scan slices (labelled A to D) demonstrating evidence of left ventricular multiple thrombi (marked by stars) A: Coronal slice with the apical inferior and anterior location of thrombi within the ventricle; B: Sagittal slice across the mid-apical location; C & D: different transverse sections demonstrating thrombi superiorly and inferiorly, respectively

The images of the CT scan (Figure 1) confirmed the presence of LV thrombi as suggested by the echocardiogram. Due to the unusual appearance on echocardiography, CT imaging was utilised to confirm the suspicion. Although this is not the most ideal modality for the assessment of such cases, it is important to note and acknowledge that this was used pragmatically here due to limitations imposed by the patient’s clinical condition.

**Figure 2:**
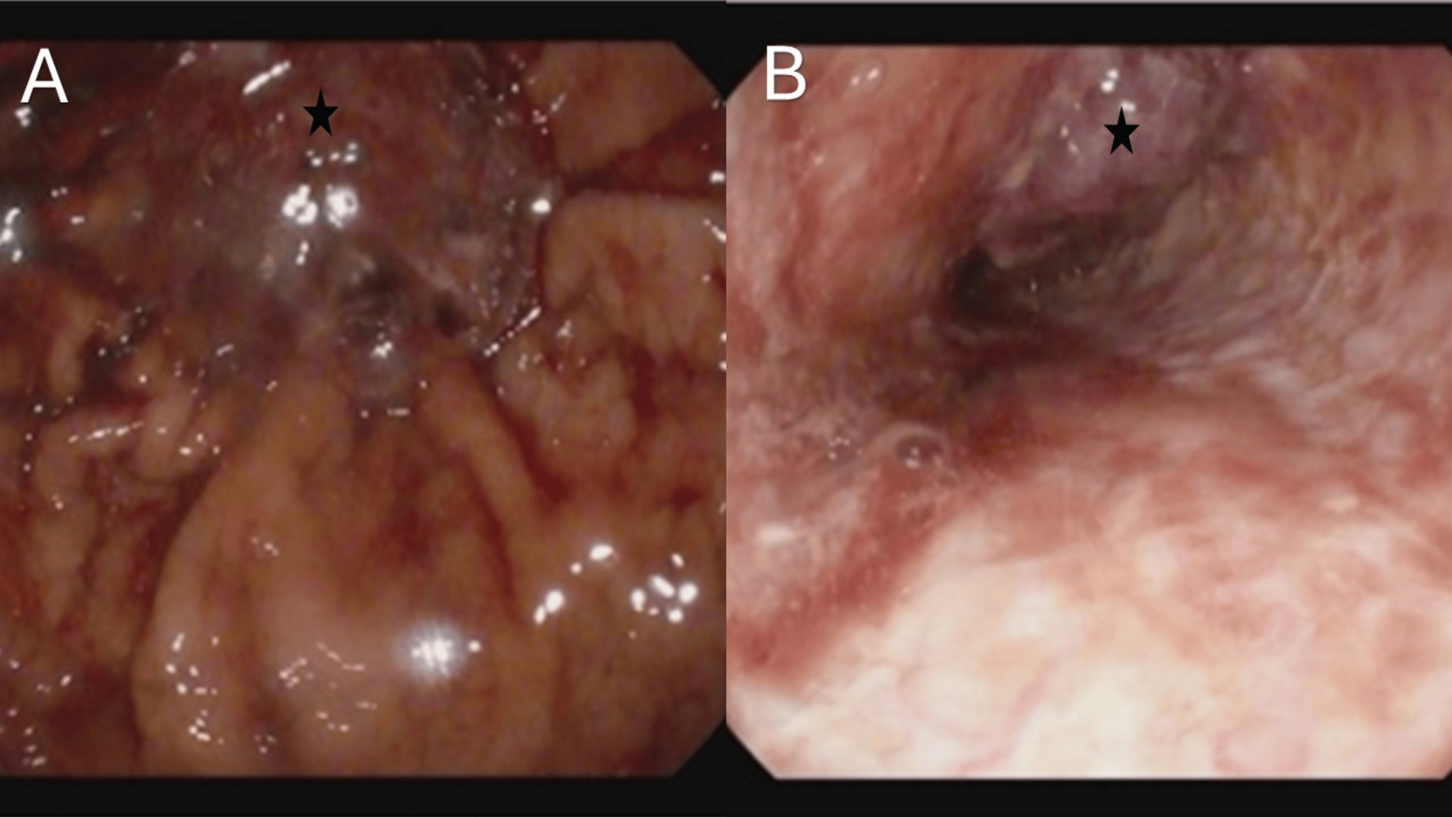
Selected upper gastrointestinal endoscopic views showing an extensive, friable, malignant-appearing oesophageal tumour extending into the cardia of the stomach A: Cardia of the stomach. Malignant-appearing tumour marked by a star; B: Middle oesophageal image 28 cm from incisors. Malignant-appearing tumour marked by a star.

On the second day of admission, the patient was noticed to have a degree of dysphagia to solid food. He was also noted to have ongoing melena. His haemoglobin plummeted from 118 g/L on admission to 96 g/L (reference range: 125-160 g/L). As the patient had originally been commenced on anti-coagulation in the form of low molecular weight heparin, this was then stopped. Due to ongoing concerns of gastrointestinal bleeding, our patient underwent an upper gastrointestinal endoscopy. Unfortunately, this revealed an extensive oesophageal, friable, easily bleeding, malignant-appearing tumour with no targets for endoscopic therapy (Figure 2). As the patient continued to deteriorate over the course of his inpatient stay, a palliative approach was deemed most plausible.

## Discussion

In cases of reduced left ventricular ejection fraction, left ventricular thrombus formation largely relates to blood stasis attributable to contractile function impairment. In such cases, there are relatively longer periods of improper flow turbulence within the ventricular cavity as compared to normal physiology. Endothelial damage is another factor that contributes to hypercoagulability [4]. Malignancy is well-known to enhance hypercoagulability as well [5]. In cases of non-ischaemic cardiomyopathy, a postulated mechanism of thrombogenesis is the myocardial inflammatory process that is associated with platelet activation [6]. Our patient suffered from severe left ventricular systolic dysfunction and was highly suspected to have had a malignant gastrointestinal neoplasm.

Thrombus formation can have one of a few outcomes: resolution, persistence and chronicity, propagation and recurrence [7-9]. As noted in our case, there was a large burden of thrombi with an echogenic structure that seems to encapsulate the thrombi on trans-thoracic echocardiography. Gao et al. described a macroscopic case of a large ball-encapsulated thrombus [10]. This patient underwent excision largely due to the diagnostic dilemma. It is noted that the Gao et al. case was identified after one year of presentation of myocardial infarction, which is in chronicity. To date, there seems to be no clear explanation of such phenomena; however, speculated mechanisms include endothelialisation, intramyocardial mural platelets activation and subsequent subacute/chronic inflammatory changes [11-12]. In general, long-term mural thrombi tend to calcify, become laminated and compact and eventually internalise beneath the endocardium, and this could represent a marker of chronicity [13]. Yamashita et al. presented a similar case in which an encapsulated tumour growth was noted to be filled with a thrombus [14].

Due to the above, we believe that encapsulation might represent an element of chronicity. However, further insights are required via further clinical and pathological studies of this phenomenon. Furthermore, more studies are needed to ascertain optimum management options whether of a medical or interventional nature.

## Conclusions

Encapsulation of left ventricular thrombosis has only rarely been reported in the literature. Our case demonstrates such a phenomenon with echocardiographic appearance. The pathogenesis remains unclear and unknown. Given the rarity, there are no insights so far on the potential management options. It also remains unclear whether encapsulation provides protection against embolization and whether clinical presentations vary accordingly.

It is also worth noting that our patient had a hypercoagulable state due to having a malignant process. Thromboembolism might have also related to his atrial fibrillation. It is unclear whether both factors might have played a role in the development of the echocardiographic appearances mentioned above. As discussed earlier, further research and data are required to guide our understanding in such cases.

## References

[1] Hooks M, Okasha O, Velangi PS, Nijjar PS, Farzaneh-Far A, Shenoy C (2021). Left ventricular thrombus on cardiovascular magnetic resonance imaging in non-ischaemic cardiomyopathy. Eur Heart J Cardiovasc Imaging.

[2] Camaj A, Fuster V, Giustino G (2022). Left ventricular thrombus following acute myocardial infarction: JACC state-of-the-art review. J Am Coll Cardiol.

[3] Turhan S, Ozcan OU, Erol C (2013). Imaging of intracardiac thrombus [Article in Czech]. Cor et Vasa.

[4] Bagot CN, Arya R (2008). Virchow and his triad: a question of attribution. Br J Haematol.

[5] Caine GJ, Stonelake PS, Lip GY, Kehoe ST (2002). The hypercoagulable state of malignancy: pathogenesis and current debate. Neoplasia.

[6] Bobbert P, Weikert U, Schmidt-Lucke C (2014). Platelet activation and thrombus formation relates to the presence of myocardial inflammation in patients with cardiomyopathy. J Cardiol.

[7] Stratton JR, Nemanich JW, Johannessen KA, Resnick AD (1988). Fate of left ventricular thrombi in patients with remote myocardial infarction or idiopathic cardiomyopathy. Circulation.

[8] Oh JK, Park JH, Lee JH, Kim J, Seong IW (2019). Shape and mobility of a left ventricular thrombus are predictors of thrombus resolution. Korean Circ J.

[9] Yeung W, Sia CH, Pollard T (2021). Predicting mortality, thrombus recurrence and persistence in patients with post-acute myocardial infarction left ventricular thrombus. J Thromb Thrombolysis.

[10] Gao C, Pan J, Tao Y, Li F (2018). Huge encapsulated thrombus secondary to myocardial infarction. Eur J Cardiothorac Surg.

[11] Frantz S, Hofmann U, Fraccarollo D (2013). Monocytes/macrophages prevent healing defects and left ventricular thrombus formation after myocardial infarction. FASEB J.

[12] Du XJ, Shan L, Gao XM (2011). Role of intramural platelet thrombus in the pathogenesis of wall rupture and intra-ventricular thrombosis following acute myocardial infarction. Thromb Haemost.

[13] Delewi R, Zijlstra F, Piek JJ (2012). Left ventricular thrombus formation after acute myocardial infarction. Heart.

[14] Yamashita C, Nakamura H, Tobe S, Koterazawa T, Yamamoto S (1992). Left ventricular diverticulum with hypertrophy of the left ventricular apex. Ann Thorac Surg.

